# Targeted validation: validating clinical prediction models in their intended population and setting

**DOI:** 10.1186/s41512-022-00136-8

**Published:** 2022-12-22

**Authors:** Matthew Sperrin, Richard D. Riley, Gary S. Collins, Glen P. Martin

**Affiliations:** 1 Division of Informatics, Imaging and Data Science, Faculty of Biology, Medicine and Health, University of Manchester, Manchester, UK; 2 Institute of Applied Health Research, College of Medical and Dental Sciences, University of Birmingham, Birmingham, UK; 3 Centre for Statistics in Medicine, Nuffield Department of Orthopaedics, Rheumatology and Musculoskeletal Sciences, University of Oxford, Oxford, UK

**Keywords:** Clinical prediction model, Validation, Generalisability

## Abstract

Clinical prediction models must be appropriately validated before they can be used. While validation studies are sometimes carefully designed to match an intended population/setting of the model, it is common for validation studies to take place with arbitrary datasets, chosen for convenience rather than relevance. We call estimating how well a model performs within the intended population/setting “targeted validation”. Use of this term sharpens the focus on the intended use of a model, which may increase the applicability of developed models, avoid misleading conclusions, and reduce research waste. It also exposes that external validation may not be required when the intended population for the model matches the population used to develop the model; here, a robust internal validation may be sufficient, especially if the development dataset was large.

## Background

Clinical prediction models (CPMs) calculate risk of current (diagnostic) and future (prognostic) events for individuals [1]. For example, QRISK calculates 10-year risk of cardiovascular outcomes [2], and EuroSCORE calculates risk of in-hospital mortality following major cardiac surgery [3]. The traditional pipeline for CPM production begins with model development, including internal validation; this is followed by external validations of the model’s performance in different data; the model’s impact may then be tested (e.g., whether its use improves health outcomes), and if considered suitable, the model may be implemented. This pipeline applies equally whether models are developed using AI or machine learning techniques, or regression-based models.

Internal validation is an examination of model performance in the same dataset that was used to develop the CPM. It is important that internal validation corrects for *in-sample optimism*, which is the tendency of models to overfit (perform better in) the development data compared with other data from the same population. This is ideally done using cross-validation or bootstrapping, but is also commonly done by splitting the dataset into training and validation subsets. For example, in the development and internal validation of a prognostic model for muscle injury in elite soccer players, an apparent c-index (a measure of the model’s ability to distinguish cases from non-cases, where a value of 1 is perfect, and 0.5 is no better than chance) of 0.64 reduced to 0.59 when using bootstrapping for optimism adjustment [4].

In contrast, external validation is an examination of model performance in different dataset(s), often regarded as a gold-standard of model ‘credibility’. Selection of the dataset(s) is critical, because model performance is highly dependent on the population and setting [5, 6]. Here, *population* refers to the group of individuals under consideration—e.g., people of a certain age, people in a specific country, people who suffer from a particular disease (and any combinations thereof). *Setting* refers to the place in which the CPM would be used, such as in hospital, primary care, general population, etc. Accordingly, there are at least three types of external validation studies. The first is where researchers investigate model performance in *one particular* population and setting carefully chosen to match the intended use of the model. This might be the same as (or similar to) the population/setting used for model development (assessing reproducibility), or might be a different population/setting (assessing transportability, e.g., evaluating if a model developed for adults has predictive value in children). A second type is where researchers investigate model performance across *multiple* populations and settings, where each is relevant to the intended use (assessing generalisability) [7, 8]; for example, in an individual patient data meta-analysis of performance across multiple countries, with a focus on identifying heterogeneity in performance [5]. In these first two types, the validation dataset(s) match the target population(s) and setting(s) where the CPM is intended for deployment, so the validation is meaningful (provided the methodological quality is also high). A third type is where researchers examine model performance in a new, conveniently available dataset, which is neither representative of the population nor the setting of interest. For example, in a comprehensive review of COVID-19 external validation studies, 35 studies were found to be at high risk of bias in the participant/data domain, which reflects the use of inappropriate dataset(s) for external validation [9]. In these cases, the validation dataset bears little relevance to any target population and setting, and thus the findings have the potential to mislead.

The aim of this paper is to describe why it is necessary to validate a CPM in a population and setting that represents each intended target population and setting of the CPM, and in a manner that reflects each intended use. These populations and settings need to be clearly reported in every validation study. We use the term *targeted validation*, which emphasises that how (and in what data) to validate a CPM should depend on the intended use of the model.

## Targeted validation

When a CPM is developed, it should be done so with a clearly defined *intended use* and *population*: i.e., when predictions are to be made, in whom, and for what purpose. Validation should be carried out to show how well the CPM performs at that specific task—a targeted validation. A focus on targeted validation has several advantages. First, a targeted validation study provides estimates of predictive performance for the intended target setting, so are extremely informative for that setting. Second, the CPM may be (perhaps subsequently) used in many clinical settings and populations—each of which may require its own targeted validation. For example, EuroSCORE was developed to predict risk of in-hospital mortality following major cardiac surgery [3, 10], but validation studies have examined if it could be used in other cardiac surgical interventions [11, 12], i.e., a different population *and* setting. For any given setting, one can assess if existing validation studies sufficiently capture the new intended use(s)/population(s), or if further validations are required. Similarly, where populations that do not match the target are used for validation, the differences can be highlighted as a ‘validation gap’ to be acknowledged or addressed (see “Validation gap” section below). Third, it focuses intention on developing and validating models that have clearly defined uses in practice, since the intended use needs to be defined a-priori, thereby avoiding research waste.

To motivate this idea, consider the following example (see Table 1). A CPM called T-MACS was developed for the prediction of acute myocardial infarction in patients presenting to the emergency department with chest pain [13]. Initially, suppose the intended use of the CPM is to aid clinical decision-making within Hospital A in Manchester, UK. The targeted validation should assess how well the model performs in (a representative sample of) patients from Hospital A, and not how the model generalises to other hospitals [15, 16]. Subsequently, suppose Hospital B in London, UK, wishes to implement the CPM; a new targeted validation should be undertaken to estimate model performance in Hospital B. The CPM has not changed but the intended target population has, hence the required validation is different.

**Table 1:** Consider T-MACS—a CPM developed for the prediction of acute myocardial infarction in patients presenting to the emergency department with chest pain [13]. Suppose our intended use is initially for hospitals within the Greater Manchester (UK) area, and then we are considering rolling out the CPM across the UK

External validation where…	Example
…a particular population and setting of intended use is of interest	T-MACS was developed using data from a hospital in Manchester, and validated using data from other hospitals in Manchester [13]. Hence the original development and validation results match the intended use. If, however, we wished to use T-MACS in London, UK, the validation above would be of limited value. We would need a new targeted validation to examine performance in London, UK.
…multiple populations and settings of intended use are of interest	Suppose we wished to implement T-MACS across all hospitals in the UK. Then, validation would be required across many hospitals in the UK, potentially using individual patient data meta-analysis of performance across hospitals to evaluate heterogeneity in performance [5]. Such a validation could also be useful to indicate expected performance of T-MACS in UK hospitals not included in the validation set. This is because, if heterogeneity in performance is low across the included hospitals, this gives some confidence that the CPM will perform well in all areas in the UK.
…an arbitrary dataset is used without consideration of the intended population or setting	A further validation was conducted in hospitals in Australia and New Zealand [14]. For our intended use, this validation offers little evidence. However, it would be very valuable if we were considering using the model in Australia and New Zealand.

Different targeted validation exercises are important because performance in one target population gives little indication of performance in another [6]. Indeed, performance is likely highly heterogeneous across populations and settings [5], due to differences in case mix (i.e., the distributions of the patient characteristics in the population), baseline risk, and predictor-outcome associations. Therefore, any discussion of validity must be in the context of the target population and setting. It is incorrect to refer to a model as ‘valid’ or ‘validated’ in general—we can only say that a model is ‘valid for’ or ‘validated for’ the particular populations or settings in which this has been assessed. Targeted validation addresses this by *first* identifying the population and setting where a model is intended to be used, and *second* identifying suitable datasets for validations that match the intended population and setting. In addition to avoiding acting on potentially misleading validation studies, a focus on targeted validation will also reduce research waste, since being explicit about the target population and use will avoid uninformative studies being conducted. To be concrete, a validation study should not take place unless a population and setting has been identified in which the CPM could potentially be used, and the validation study should be designed to estimate performance in *that* population and setting.

This is not a new idea: we are simply making it explicit. Riley et al. [5], state ‘external validation uses new participant level data, external to those used for model development, to examine whether the model’s predictions are reliable (that is, accurate enough) in individuals from potential population(s) for clinical use’ while Wessler et al. [6], remark that we should not accept a CPM in a particular context ‘unless CPM performance is specifically known to be excellent in populations like those’. It is also emphasised in the PROBAST risk of bias tool for systematic reviews and meta analyses of CPMs, where the ‘applicability’ domain checks whether included studies consider the same setting and population as the review question [17], emphasised in a recent scoping review of guidance for prediction models using AI [18], and included in the protocol for reporting and risk of bias tools for prediction models developed using AI [19]. *Target validity* in the clinical trial literature, which quantifies bias in transporting a trial-estimated causal effect to a target population, has a similar motivation [20].

Moreover, the targeted validation framework suggests that there may be contexts where the data used for validation could be the same as for development. In the first part of the example above, the closest data to the intended target population (Hospital A) may be the development data (Table 1). In this case, there is little to be gained through evaluating performance in other hospitals; instead, the focus should be on a thorough internal validation using the development dataset. This internal validation is likely to give a robust estimate of the model’s performance when appropriate steps were taken during the model development to ensure the study has adequate sample size [21], that overfitting is minimised [22], that in-sample optimism is estimated precisely and corrected for [8], and that the optimism was examined by replaying all the model development steps. Moreover, the internal validation should include, for example, temporal or demographic subgroups to test the reproducibility and generalisability of the model. Provided all these steps are thoughtfully conducted, internal validation can be viewed as a reliable measure of performance in the intended population, and the lack of any external validation is not a concern. Indeed, whenever a new model is developed, the model development data should always be chosen according to the anticipated target population and setting: for example, if a model is intended to be used in UK primary care, then UK primary care data should be used to develop the model.

## One size fits all versus tailored models

Consider the situation where we wish to implement T-MACS across all hospitals in the UK (Table 1). Here, we could evaluate the CPM in each hospital, and then—depending on the observed performance, and a subsequent impact assessment study—choose to either deploy the model as originally specified, or deploy it after updating it for each particular context [23, 24]. This situation—in which one wishes to implement a CPM across multiple populations/settings—is common, and there are two main ways of achieving this: building a single CPM for use in all target settings or building tailored CPMs for each target setting.

Under the first approach, one needs to assess generalisability of the CPM [25–27]. A natural way of doing this is to obtain (new) datasets from multiple populations (e.g., across countries, or across clusters of data within electronic health records), evaluate performance of the relevant model in each dataset/cluster, and then meta-analyse [28], with particular attention to quantifying and identifying sources of heterogeneity [5]. Alternatively, we might conduct internal-external cross-validation to combine model development with assessment of model generalisability across the multiple populations/settings [5, 8, 29].

However, developing a model that generalises across multiple populations is difficult, not least because predicted risks are unlikely to calibrate well with observed risks in every population and setting. Methods are emerging that support this [30], and incorporating causal inference principles is also likely to help generalisability and transportability [31, 32]. Nevertheless, insisting on a model with broad general applicability comes at the price of reduced performance in specific settings or populations [15]. Model performance being worse in specific subgroups also raises concerns over fairness [33]. As such, the second approach—in which one starts with a CPM developed using sufficient (and appropriate) data [21], and then tailors or updates it to local settings [23, 24]—may be appealing. This implies *targeted updating* of a given CPM updated for specific target populations/settings; following this, targeted validation exercises would be needed in each local population/setting to examine the locally tailored CPM. However, the feasibility of having a large family of tailored CPMs is a challenge with regard to provenance and maintenance.

## Validation gap

Focus on targeted validation makes the interpretation of the predictive performance clearer. If the target population is patients in Hospital B, then we need to estimate model performance in Hospital B. If we can obtain data, and have the resource, to validate the model in this population, then the corresponding performance estimates are appropriate. However, if the validation had instead been performed in Hospital C (for example, if there is little or inadequate historical data available in Hospital B, or resource constraints do not permit the validation study to be conducted in Hospital B), then targeted validation allows one to infer how applicable the predictive performance estimates we obtain might be for Hospital B given the difference between the two settings: a ‘validation gap’. Identification of a validation gap suggests caution in using the CPM within the target population. In this situation, we recommend that differences between the validation population and target population can be described qualitatively, such as by contrasting the setting, case-mix and patient eligibility criteria; or quantitatively (where sufficient data exists), by examining membership models for whether individuals belong to the validation population or target population [25, 34]. We then recommend being explicit about the required assumptions for the validation results to transport, and to address the differences where possible, such as through reweighting the validation population to resemble the target population [35]. This reweighting could be done at individual level, or at group level—for example, if performance is known to vary across groups of patients in different disease subgroups, then performance in each of the subgroups could be reported in the validation population, then combined through appropriate weighting to estimate performance in the target population. Such reweighting would allow estimation of global performance measures such as AUC, while the weights themselves can be used to infer where the differences between the validation and target are largest (e.g., under-represented subgroups), and therefore where the CPM may have poor local performance in the target population (i.e., issues with strong calibration as defined in [36]). The validation gap concept can also help researchers to decide when a full new validation exercise might not be necessary—i.e., where existing validations are performed in sufficiently similar populations and settings, and the model has been shown to be generalisable.

A particular challenge that targeted validation emphasises is that the implementation of the CPM will always be *after* validation and subsequent impact study—so a validation gap in time will always be present [37]. CPMs can be prone to changes in the underlying distribution, which causes calibration drift and other performance issues, particularly in contexts such as surgical risk [38], and infectious disease risk [39]. Therefore, model development strategies that allow a CPM to respond to changes over time—such as dynamic modelling [40, 41] or temporal recalibration [42]—are very promising. This also emphasises the importance of a validation exercise thoroughly considering heterogeneity in geography, over time, and over setting [5], and indeed the possibility of targeted updating, in which CPMs are updated to a new time period before revalidating.

## Conclusion

We recommend that validation of clinical prediction models should relate to the target population and setting, and suggest using the term targeted validation to make this focus explicit. This provides a framework in which researchers are transparent about the intended use of the model being validated, and motivates the use of a validation dataset that is representative of the population(s) and setting(s) of intended use. If such a dataset is not available, and validation is undertaken, then researchers should highlight differences between the validation and target populations (a ‘validation gap’) so that the findings can be placed in context.

There are three key implications of focusing on targeted validation. First, validation studies that do not state, and clearly justify, the intended target population or setting are not fit for purpose. The prevalence of this problem has not yet been quantified. Second, a new intended use of a model requires a new targeted validation exercise (which may be a new validation, or careful consideration of the relevance of existing validations): CPMs should not be referred to as ‘valid’ or ‘validated’ as this is meaningless without reference to a target population. Third, external validation studies may not always be needed, specifically if the development dataset is sufficiently large, already represents the target population and setting, and appropriate steps have been taken to adjust performance estimates for in-sample optimism.

## Data Availability

No data were generated or analysed in support of this research.

## Abbreviations

CPM: Clinical prediction model
EuroSCORE: European system for cardiac operative risk evaluation
T-MACS: Troponin-only Manchester acute coronary syndromes decision aid
